# tRNA modifications: greasing the wheels of translation and beyond

**DOI:** 10.1080/15476286.2024.2442856

**Published:** 2024-12-26

**Authors:** Minjie Zhang, Zhipeng Lu

**Affiliations:** a Key Laboratory of Breast Cancer Prevention and Therapy, Tianjin Medical University, Tianjin, China; b Tianjin Key Laboratory of Medical Epigenetics, Department of Bioinformatics, School of Basic Medical Sciences, Tianjin Medical University, Tianjin, China; c Department of Pharmacology and Pharmaceutical Sciences, University of Southern California, Los Angeles, CA, USA; d Norris Comprehensive Cancer Center, University of Southern California, Los Angeles, CA, USA; e Eli and Edythe Broad CIRM Center for Regenerative Medicine and Stem Cell Research, University of Southern California, Los Angeles, CA, USA

**Keywords:** tRNA modifications, tRNA-modifying enzyme, tRNA modification functions, detection technologies, RNA modopathies

## Abstract

Transfer RNA (tRNA) is one of the most abundant RNA types in cells, acting as an adaptor to bridge the genetic information in mRNAs with the amino acid sequence in proteins. Both tRNAs and small fragments processed from them play many nonconventional roles in addition to translation. tRNA molecules undergo various types of chemical modifications to ensure the accuracy and efficiency of translation and regulate their diverse functions beyond translation. In this review, we discuss the biogenesis and molecular mechanisms of tRNA modifications, including major tRNA modifications, writer enzymes, and their dynamic regulation. We also summarize the state-of-the-art technologies for measuring tRNA modification, with a particular focus on 2’-O-methylation (Nm), and discuss their limitations and remaining challenges. Finally, we highlight recent discoveries linking dysregulation of tRNA modifications with genetic diseases.

## Introduction

Transfer RNA (tRNA) is the key adaptor molecule in protein synthesis, facilitating the accurate translation of genetic information from messenger RNA (mRNA) into proteins [1]. tRNA molecules typically range from 70 to 90 nucleotides, and fold into clover-leaf secondary structures and L-shaped tertiary structures. Each tRNA molecule carries a specific amino acid at the 3’-end to the elongating peptide chain in the ribosome. A recent review by Phizicky and Hopper provides an in-depth exploration of tRNA’s structural dynamics, maturation process, and functional roles [2].

tRNA molecules undergo extensive chemical modifications [3,4]. To date, more than 170 RNA chemical modifications have been discovered in RNAs [5,6], about half of which are present in tRNAs, including methylation, pseudouridylation, acetylation, deamination and thiolation, etc. Chemical modifications in the structural core of tRNAs are required for their stability and functional folding [7,8], whereas modifications at or near the anticodon loop (34-35-36) directly regulate decoding. In particular, modifications at positions 34 (wobble) and 37 (3’ of the anticodon triplet) stabilize the codon-anticodon base pairing, especially the wobble pairing, and maintain the reading frame by forming additional base-stacking interactions (see review [9]).

In bacteria, each tRNA molecule carries an average of eight modifications, while each eukaryotic tRNA typically carries about 13 modifications [10]. The increasing chemical complexity of tRNA modifications parallels many other molecular mechanisms in evolution, e.g. gene number, regulatory DNA/RNA elements, and epigenetics, underlying the increasing biological complexity in the tree of life. While the basic structure of tRNA has remained nearly unchanged during the billions of years of evolution due to the nearly irreducible constraints of the translation machinery, a large number of chemical modifications have emerged to ‘grease’ the wheels of life, making it possible to accommodate exquisite regulation in multicellular organisms.

Recent developments in new modification mapping technologies, discoveries of reversible RNA modifications, and their connections to human diseases have renewed interests in the diverse chemistry and functions of tRNA epi-transcriptomes. In the last few years, several classes of advanced technologies have been developed to study the presence, quantity, and dynamics of tRNA modifications, such as chemical/antibody detection and next-generation sequencing (NGS), nanopore direct RNA sequencing, and mass spectrometry [11]. To date, hundreds of tRNA modification enzymes have been discovered [12]. Aberrant expression or dysfunction of tRNA modifying enzymes causes a variety of human diseases, such as neuro-developmental disorders, mitochondrial disorders, metabolic diseases and cancers [10,13,14]. However, the basic mechanisms and disease aetiology of most tRNA modifications are poorly understood.

In this review, we provide a historical perspective on tRNA modifications, and then focus on the recent advancements on the discovery and mechanistic studies of tRNA modifications, as well as the development of new mapping technologies. In the end, we also discuss the association of tRNA modifications with human genetic diseases. This review aims to provide a broad overview of the field and highlight state-of-the-art technologies for measuring tRNA modifications. Rather than presenting a comprehensive summary, we point readers to other recent reviews for more in-depth coverage of specific topics on tRNA modifications and their biology.

## Brief history of tRNA modification research

**Figure 1.**
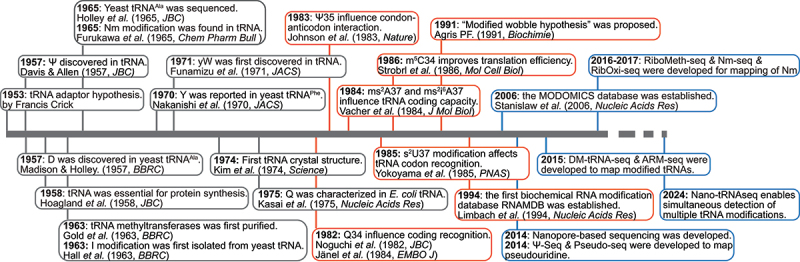
Timeline of significant discoveries in the field of tRNA modification. The first tRNA modification pseudouridine (Ψ) was discovered in 1957. Since then, various modifications such as I, D, Y, yW and Q were also discovered. With the accumulation of epi-transcriptomic knowledge, comprehensive databases like RNAMDB and MODOMICS were established to catalogue these modifications. While earlier studies primarily used MS for modification quantification, high throughput mapping methods gradually emerged in the last decade for mapping different types of tRNA modifications at nucleotide resolution. The black boxes in the timeline indicate discoveries before the 1980s, mainly focusing on identifying tRNA modifications. The red boxes cover advancements between the 1980s and 1990s, emphasizing how modifications affect tRNA structure, codon recognition, and translational efficiency and fidelity. The blue boxes represent discoveries from 2000 to the present, highlighting new technologies for detecting tRNA modifications and their roles in human diseases, drug resistance, and cellular metabolism.

Modified nucleosides in RNA, beyond canonical adenosine (A), uridine (U), cytidine (C) and guanosine (G), have been recognized for more than half a century. Figure 1 illustrates the historical milestones of tRNA modification research. In the early 1950s, the existence of tRNA was first hypothesized by Francis Crick as an adaptor molecule in protein synthesis [15]. In 1958, Hoagland and Zamecnik provided the first experimental evidence that tRNA molecules carry activated amino acids for polypeptide synthesis [16].

Pseudouridine (Ψ) was first identified in yeast tRNA and then shortly after in bacterial tRNA [17–19] and termed the ‘fifth nucleotide’ due to its relative abundance after A, U, C and G [20]. Following the discovery of Ψ, significant efforts were made in the 1960s and 1970s to identify other ‘rare’ nucleotides in tRNAs and their corresponding enzymes. For example, Gold M. and colleagues first purified RNA methyltransferases between 1963 and 1966, including those that methylate tRNA [21–24]. The presence of biological 2’-O-methylribose nucleotides (Cm, Gm, Um and Am) was confirmed in tRNAs by Furukawa and others, highlighting the diversity of tRNA modifications [25–27]. In 1965, Holley and his team sequenced the alanine tRNA from yeast and confirmed 10 more modifications on tRNAs, including inosine (I), dihydrouridine (D), 1-methyladenosine (m^1^A) and 5-methylcytidine (m^5^C) [28].

In the 1970s, significant advancements were made in identifying hypermodified nucleosides in tRNAs, such as wybutosine (yW) and queuosine (Q). Nakanishi et al. first reported the identification of Y base in yeast phenylalanine tRNA (later renamed wybutosine, or yW) [29–31]. In 1975, the modified nucleoside queuosine (Q) was structurally characterized in *Escherichia coli* (*E.* *coli*) tRNA^Tyr^, tRNA^His^, tRNA^Asn^ and tRNA^Asp^, using mass spectrometry, proton nuclear magnetic resonance, and ultraviolet absorbance analysis. Following its initial characterization in prokaryotes, Q was subsequently detected in eukaryotic tRNAs, highlighting the evolutionary conservation and functional importance of Q modification [32]. In 1976, Aschhoff identified the enzyme responsible for 7-methylguanine (m^7^G) modification in *E.* *coli* tRNA [33].

Studies in the 1980s and 1990s began to explore the roles of tRNA modifications in maintaining tertiary structure and facilitating codon-anticodon pairing during translation [34–38]. For example, Q was found at position 34 in certain tRNAs, which enhances the efficiency and accuracy of codon recognition. In *E.* *coli*, a mutant lacking Q34, known as the tgt mutant, is unable to survive in the stationary phase and fails to synthesize nitrate reductase under anaerobic conditions [39,40]. Additionally, in 1986, Strobel et al. discovered that the m^5^C34 modification in *S.* *cerevisiae* tRNA also enhances translation efficiency [41]. Like position 34, the nucleoside at position 37 is often modified and plays a crucial role in regulating the translation efficiency and fidelity [42,43]. The modified wobble hypothesis was proposed in 1991, introducing the idea that specific modifications on tRNA wobble position can shape the anticodon architecture and emphasizing the role of post-transcriptional modifications in tRNA function [35,44].

In the 2000s, tRNA modifications were found to be dynamically regulated in response to the levels of cellular metabolites and altered in human diseases [13,45–49]. From the 2010s to the present, the development of advanced technologies such as mass spectrometry and NGS has enabled a more comprehensive and quantitative mapping of modifications on tRNAs, leading to a better understanding of their diversity and functions [50–59]. Here, we summarize the current knowledge of tRNA modifications, focusing on the regulatory mechanisms, biological consequences, and disease relevance of several well-studied tRNA modification types.

## tRNA modification chemistry

**Figure 2.**
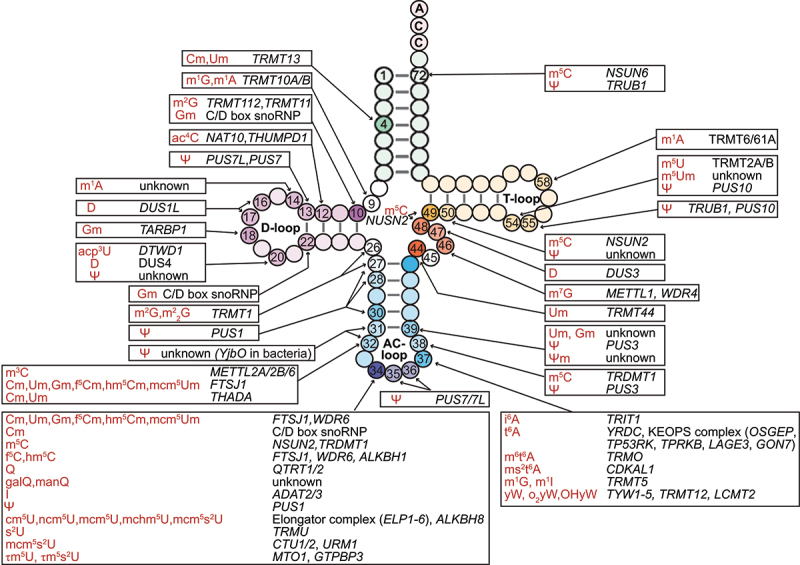
Schematic representation of human cytoplasmic tRNA modifications and their writer enzymes. Modified nucleotides are numbered. Each type of tRNA modification and their modifying enzymes are indicated. Modification enzymes that are confirmed in other organisms are listed in parentheses. The abbreviation of each RNA modification conforms with the RNA modification database MODOMICS^6^. Um (2’-O-methyluridine), Gm (2’-O-methylguanosine), Cm (2’-O-methylcytidine), m^2^G (N2-methylguanosine), m^1^G (1-methylguanosine), m^1^A (1-methyladenosine), ac^4^C (N4-acetylcytidine), Ψ (pseudouridine), D (dihydrouridine), acp^3^U (3-(3-amino-3-carboxypropyl)uridine), m^2^_2_G (N2,N2-dimethylguanosine), m^3^C (3-methylcytidine), f^5^Cm (5-formyl-2’-O-methylcytidine), hm^5^Cm (2’-O-methyl-5-hydroxymethylcytidine), mcm^5^Um (5-methoxycarbonylmethyl-2’-O-methyluridine), m^5^C (5-methylcytidine), f^5^C (5-formylcytidine), hm^5^C (5-hydroxymethylcytidine), Q (queuosine), galQ (galactosyl-queuosine), manQ (mannosyl-queuosine), I (inosine), cm^5^U (5-carboxymethyluridine), ncm^5^U (5-carbamoylmethyluridine), mcm^5^U (5-methoxycarbonylmethyluridine), mchm^5^U (5-(carboxyhydroxymethyl)uridine methyl ester), mcm^5^s^2^U (5-methoxycarbonylmethyl-2-thiouridine), s^2^U (2-thiouridine), τm^5^U (5-taurinomethyluridine), τm^5^s^2^U (5-taurinomethyl-2-thiouridine), i^6^A (N6-isopentenyladenosine), t^6^A (N6-threonylcarbamoyladenosine), m^6^t^6^A (N6-methyl-N6-threonylcarbamoyladenosine), ms^2^t^6^A (2-methylthio-N6-threonylcarbamoyladenosine), m^1^I (1-methylinosine), yW (wybutosine), o_2_yW (peroxywybutosine), OHyW (hydroxywybutosine), ψm (2’-O-methylpseudouridine), m^7^G (7-methylguanosine), m^5^Um (5,2’-O-dimethyluridine).

RNA molecules undergo a wide variety of chemical modifications, including, but not limited to, methylation, pseudouridylation, thiolation, isomerization, deamination, hydroxylation, and ribose 2’-O-methylation [10]. To date, more than 100 species of chemical modifications have been characterized in tRNAs [60,61]. The specific incorporation of tRNA modifications along the tRNA sequence adds an additional layer of tRNA complexity, contributing to the vast combinatorial diversity of tRNA species and aiding in maintenance of proper folding and structural integrity of tRNAs [8] (Figure 2). Recently, Suzuki has provided an expanded and integrated view of tRNA modification chemistry [48]. In the following sections, we introduce several common and recently discovered tRNA modifications.

Methylation is the most common base modification in tRNA [62–64]. All four nitrogenous bases undergo methylation either at nitrogen (N) or carbon (C) atoms, such as 1-methyladenosine (m^1^A), 3-methylcytidine (m^3^C), 5-methylcytidine (m^5^C), N^2^-methylguanosine (m^2^G), N^2^,N^2^-methylguanosine (m^2^_2_G), N^6^-methyladenosine (m^6^A) and 7-methylguanosine (m^7^G) (reviewed in [65]). The addition of methyl groups to tRNA nucleotides is catalyzed by different methyltransferases, including the TRM (tRNA methyltransferase) family [66], the NSUN family [67,68], and the METTL family [69,70]. Each methyltransferase exhibits substrate specificity for particular tRNA sequences and target nucleotides. The ALKB family members catalyse a variety of demethylation reactions [71,72]. Methylation of the ribose sugars at the C2’-position (Nm) in tRNA molecules is catalysed by either the TRM family or by Fibrillarin (FBL), guided by C/D box small nucleolar RNAs (snoRNAs) (Figure 2) [13,66,73,74]. The 5-methyluridine modification at position 54 (m^5^U54) is one of the most common and conserved tRNA modifications [75]. This modification is catalyzed by tRNA methyltransferase Trm2 in eukaryotes and TrmA in bacteria. m^5^U54 plays a crucial role in stabilizing the tRNA tertiary structure and preventing the formation of tRNA-derived small RNAs (tsRNAs) [76,77].

Pseudouridylation (Ψ) is the most abundant and prevalent modification in tRNA. Unlike other modifications that add or remove functional groups, pseudouridine is an isomer of the nucleoside uridine in which the uracil is attached to the ribose via a carbon–carbon instead of a nitrogen–carbon glycosidic bond. Ψ modifications in tRNA are generated by stand-alone enzymes known as pseudouridine synthases (Pus enzymes) in eukaryotes. Rintala-Dempsey et al. provided a comprehensive overview of the different Pus enzymes and their modifying positions in tRNAs [78]. The Ψ modification at position 55 (Ψ55) in tRNA is one of the most universally conserved RNA modifications across all forms of life analysed to date. Modification of Ψ55 is catalysed by TruB in bacteria [79], Cbf5 or Pus1 in archaea [80,81], and Pus4 in eukaryotes [82,83]. Ψ plays a crucial role in maintaining tRNA structure and stability, ultimately influencing protein synthesis and cellular physiology [84–88]. Additionally, the cocrystal structure of Ψ55 modifying enzyme TruB and its homologs suggest that Pus enzymes also act as RNA chaperones, assisting in the correct folding of their substrate tRNAs [89,90].

Dihydrouridine (D) is a highly conserved modified base, typically found within D-loop of tRNAs across all domains of life [91]. D is synthesized from U by dihydrouridine synthase (Dus), with DusA, DusB and DusC being the bacterial members of this enzyme family [92,93]. The reduction of the uridine C5-C6 bond creates a saturated nonplanar and nonaromatic nucleobase. The unique biochemical specificities of D contribute to the cloverleaf-like tRNA secondary structure and play a crucial role in stabilizing the L-shaped tertiary structure by interacting with the T-loop [91]. The primary function of D is to protect tRNAs from degradation, and its levels are notably increased in tumours [91,94].

Thiolation is another highly conserved family of modifications in tRNA, including 4-thiouridine (s^4^U), 2-thiouridine (s^2^U) and 2-thiocytidine (s^2^C) (Figure 2). These sulfur-containing modifications are commonly found at seven different positions in tRNA: 8, 9, 32, 34, 37, and 54. The s^4^U is a common modified nucleoside at position 8 and 9 in tRNA from bacteria and archaea, where it is introduced by the enzyme Thil [95]. In *E.* *coli*, s^4^U acts as a near-ultraviolet (UVA) radiation sensor to protect tRNA from ultraviolet (UV) damage by absorbing UV light and reducing the formation of harmful photoproducts [96–99]. The s^2^U derivative in the form of 5-methyl-2-thiouridine (m^5^s^2^U), also known as 2-thioribothymidine (s2T), at tRNA position 54 has been reported in thermophilic bacteria, such as Thermus thermophilus. The 2-thiolation of m^5^s^2^U54 in T. thermophilus is facilitated by four factors (TtuA, TtuB, TtuC, and TtuD), and the 2-thio group of m^5^s^2^U54 contributes to the overall stability of the tRNA [100–102]. Other s^2^U derivatives, such as 5-methylaminomethyl-2-thiouridine (mnm^5^s^2^U) and 5-methoxycarbonylmethyl-2-thiouridine (mcm^5^s^2^U) at the wobble position in tRNAs (Figure 2) are catalyzed by MnmE-MnmG complex [103] and the Elongator complex [104], respectively. These modifications are critical for proper mRNA decoding and protein translation [105–108]. s^2^C at position 32 in bacterial tRNA is catalysed by TtcA [109] and plays a significant role for modulating the flexibility and conformation of the anticodon loop, avoiding the formation of the rigid U-turn structure in anticodon loops [110,111].

In the past two decades, numerous rare and novel tRNA modifications were discovered, expanding the chemical space of tRNA modifications (reviewed in [48]). For example, m^1^s^4^Ψ (1-methyl-4-thio pseudouridine) is reported to occur at the U54 position of tRNAs from Ignicoccus hospitalis [112], an archaeon that thrives in high-temperature and high-pressure environments. m^1^s^4^Ψ is present in the T-loop, contributing to the stability of the tRNA tertiary fold under extreme hyperthermic conditions [112]. Queuosine (Q) was first discovered by Nishimura in the 1970s and remains one of the most complex hypermodified nucleosides at the wobble position (position 34) of certain tRNAs [113–117] (Figure 2). Recent research has identified further modified derivatives of Q in tRNAs, where the cyclopentene ring structure is conjugated to a galactosyl-, mannosyl-, or glutamyl residue to generate galactosyl-Q (galQ), mannosyl-Q (manQ) and glutamyl-Q (gluQ) [118–120]. The sugar-modified Queuosine derivatives (galQ and manQ) are found in the tRNAs of vertebrates, while glutamylated Queuosine (gluQ) is only known in bacteria [121]. Due to space limitations, this review does not offer a comprehensive overview of all these novel modifications (see another review [48]). These newly identified modifications represent an important area of research, as their biological functions and potential roles in human health and disease are still not fully understood.

## Functional significance of tRNA modifications

A wide variety of tRNA modifications are highly conserved across various organisms, underscoring their fundamental importance. Modifications in the anticodon loop are crucial for codon–anticodon interactions and reading frame maintenance, thereby ensuring translation accuracy. The body regions of tRNAs are also frequently modified, which is fundamental to stabilizing structures and diverse biological functions of tRNA molecules. In addition to catalysing modifications, many tRNA modifying enzymes have been demonstrated to play important roles in tRNA folding and other functions [122]. In this section, we briefly introduce several examples to discuss the functions of tRNA modifications.

**tRNA modifications at anticodon**. The anticodon region is the most diversely modified in tRNA molecules. To date, more than 30 types of chemical modifications have been described at the wobble position, which contributes to the precise recognition and decoding of mRNA codons during translation. tRNA modifications at wobble position are installed by multiple different writer enzymes (e.g. NSUN2, NSUN3, ADAT2, ADAT3, CTU1, CTU2, ELP3, MTO1, QTRT1, WDR6, PUS1, FTSJ1, ALKBH8, GTPBP3 and TRMU) and one eraser enzyme (ALKBH1) (Figure 2). Most wobble modifications, such as Q, yW, mnm^5^s^2^U, and mcm^5^s^2^U, stabilize G-U wobble base pairs and prevent the misreading of near-cognate codons during translation [123]. Recent studies have shown that the loss of anticodon wobble uridine (U34) modifications in a subset of tRNAs, such as the absence of mcm^5^s^2^U modification, leads to ribosome pausing, slower translation, and increased translation errors [50].

Ψ is structurally similar to uridine but differs in its glycosidic bond configuration and H-bonding capacity at the nucleobase, allowing it to form stable base pairs with multiple nucleotides at the wobble position, including not only A and G but also, to a lesser extent, with C and U. This flexibility enables tRNA molecules to recognize multiple synonymous codons with varying efficiency [124]. In addition, modifications such as I34 (Inosine), f^5^C (5-formylcytidine) and m^5^C at wobble position extend the decoding capability of tRNAs, contributing to optimal codon-anticodon pairing, translation efficiency and accuracy [125–128].

Modifications at position 37, right after the anticodons, such as m^5^C, m^5^U, yW, Gm, t^6^A (N^6^-threonylcarbamoyladenosine) and i^6^A (Isopentenyladenosine), enhance the stability of the codon and anticodon coupling via cross-stacking with the first base of the codon [108,129,130]. These modifications can promote tRNA binding to the A-site and facilitate efficient translocation, ensuring the specificity and efficiency of codon–anticodon interactions during translation (reviewed in [9]).

**tRNA modifications outside the anticodon loop**. The nucleotides outside of the anticodon loop, including the D-loop, T-loop, variable loop, and acceptor stem, are also heavily modified. These modifications, such as base methylation (e.g. m^5^C, m^1^A), Ψ and Nm, stabilize tRNA structures by strengthening the base stacking interactions and protecting tRNA from degradation by ribonucleases. Recently, Schultz et al. found that modifications in the T arm of tRNA also play a crucial role in determining tRNA maturation, function, and cellular fitness [131]. In this section, we introduce the functions of several common tRNA modifications.

m^5^C most frequently occurs at the junction between the variable loop and the T-stem, spanning nucleotides 47–50 [132,133]. Several methyltransferases for m^5^C have been discovered, including tRNA aspartic acid methyltransferase 1 (TRDMT1, also known as DNMT2), tRNA methyltransferase NSUN and METTL families [69,134,135]. m^5^C at position 48 (m^5^C48) increases the hydrophobicity of base pairs and promotes base stacking in the tRNA, enhancing protein synthesis [69,136]. Besides C48, Goll et al. found that TRDMT1/DNMT2 specifically installs m^5^C38 in the anticodon loop of tRNA^Asp^, protecting tRNA^Asp^ from stress-induced endonuclease-mediated cleavage and promoting the translation of multiple Asp-enriched proteins [135].

m^2^G (N^2^-methylguanosine) is widespread at positions 6–10, 26 and 27 in tRNA molecules and conserved in eukaryotes, archaea, and some bacteria. The complex formed between THUMP domain-containing protein 3 (THUMPD3) and TRMT112 is responsible for m^2^G6 in a broad range of tRNAs and m^2^G7 in tRNA^Trp^ in human cells. THUMPD3 knockout cells exhibited impaired global protein synthesis and reduced growth [137]. TRMT1 is m^2^G26 methyltransferase that targets G26 position of cytoplasmic tRNAs [138]. The enzyme can also be targeted to mitochondria for the m^2^G26 modification in mitochondrial tRNAs. In higher eukaryotes, m^2^G at position 26 in most cytoplasmic tRNAs is predominantly modified again into m^2^_2_G by TRMT1 [139]. m^2^_2_G in tRNA acts as a molecular hinge, adjusting tRNA architecture and increasing its thermostability [140,141]. Loss of m^2^_2_G due to TRMT1 mutations disrupts global translation, impairs cell proliferation and causes a neurological disease [142].

m^7^G occurs at position 46 in the variable region of tRNAs that contain the ‘RAGGU’ motif. The METTL1-WDR4 complex is the methyltransferase that instals m^7^G46 in certain tRNAs [143,144]. m^7^G46 modification forms a triplet with C13-G22, contributing to the integrity and stability of a large subset of tRNAs [145–147]. m^7^G tRNA modification selectively regulates the translation of oncogenic transcripts, such as the genes related to cell-cycle and epidermal growth factor receptor (EGFR) pathway [148]. Loss of METTL1 results in decreased m^7^G tRNA methylation, which then alters the cell cycle and inhibits oncogenicity in numerous cancer types [149–152]. In addition, mutations in WDR4 also cause several human neurodevelopmental defects, including Galloway-Mowat syndrome (GAMOS) and microcephaly [153–155].

Nm is a common RNA modification consisting of the methylation of the ribose 2’-OH moiety. Nm can occur at any of the four nucleotides, as well as in combination with other modified nucleotides (e.g. m^6^Am at the 5’ end of mRNAs, and many combinations in tRNAs, Figure 2), explaining the abundance of this modification [156,157]. In *E.* *coli* tRNAs, the major Nm residues are located in the D-loop at position 18 (Gm18) and the anticodon loop at positions 32 (Cm32) and 34 (Nm34) [158,159]. In eukaryotic tRNAs, in addition to the conserved Gm18, Cm32 and Nm34 residues, several other positions are also 2’-O-methylated: Nm4, Nm44 and Nm54 [157]. From bacteria to humans, Nm modifications on tRNAs have evolved from a simple site to a more complex pattern. Nm residue in eukaryotic tRNAs often exists as part of a more complex modified nucleotides, where base modification is coupled with ribose methylation (e.g. m^5^Um, Ψm, f^5^Cm, ncm^5^Um and m^1^Am) [160–169].

Additionally, Nm in archaea and eukaryotic tRNAs can also be introduced by the structurally and functionally conserved small nucleolar RNA-protein complexes (C/D box snoRNPs) [73,74]. This mechanism was first reported in archaea more than 20 years ago [170,171]. Recently, Vitali and Kiss demonstrated that snoRNAs SNORD97/SNORD133 (D97/D133) guide Cm34 for the elongator Met tRNA [73]. Using PARIS2, a recently developed technology for high-throughput RNA–RNA interaction mapping [172], and dRMS, an improved method for Nm mapping [74], we identified an extensive snoRNA–tRNA network that targets nearly all nuclear-encoded tRNAs, suggesting a much broader impact of snoRNAs in tRNA Nm modifications [74]. Functionally, Nm modification increases the stability of tRNA against alkaline/enzymatic hydrolysis and environmental stress [158]. Moreover, Nm modifications in tRNAs are also involved in translational regulation and innate immune evasion. For example, Gm18 in *E.* *coli* tRNA acts as an effective structural anti-determinant for RNA recognition by the mammalian innate immune system via Toll-like receptors (TLR7), even though this modification cloak does not effectively block recognition of whole bacterial organisms [173–176].

Other common tRNA modifications outside the anticodon loop include m^5^U at position 54 [76,77], Ψ at position 55 [89], m^1^A at positions 9 and 58 [177], and D modifications within D-loop of tRNAs [91]. These well-characterized modifications play crucial roles in maintaining tRNA stability, structural integrity, and proper folding. Beyond their roles in tRNA stability, modifications outside the anticodon loop are also implicated in several essential cellular functions, such as translation, fitness, and stress adaptation [10,178]. For example, the m^2^_2_G26 modification, located between the D and anticodon stems, increases the stiffness of the modified tRNA, facilitating its conformational dynamics during ribosomal translocation in elongation [140,179,180]. Additionally, Jones et al. found that m^5^U54 plays a crucial role in tRNA maturation and ribosome translocation during protein synthesis [181]. Moreover, Saleh and Farabaugh used *in* *vivo* mistranslation reporters to show that many tRNA core modifications modulate the frequency of misreading errors, depending on the identity of the tRNA and the codon being read [182]. Recent studies have increasingly focused on characterizing the distinct roles of these modifications that extend beyond their contributions to tRNA stability [178].

**Modification circuits in tRNAs**. Numerous tRNA modifications play critical roles for their functions. While most modifications are introduced to tRNA independently, a dynamic adaptation of the modification status of tRNAs has been identified, where one or more modifications stimulate or regulate the formation of another. The majority of the modification circuits are predominantly found in the anticodon loop region (reviewed in [183]). For example, Cm32 and Gm34 facilitate the formation of yW37 in tRNA^Phe^ of *S.* *pombe*, *S.* *cerevisiae*, and humans [168,184,185]. i^6^A37 promotes m^3^C32 formation in tRNA^Ser^ of *S.* *pombe* and *S.* *cerevisiae* [186,187], and t^6^A37 drives m^3^C32 formation in tRNA^Thr^ of *S.* *cerevisiae* [187]. In *E.* *coli*, i^6^A37 drives Cm34 and Um34 formation in tRNA^Leu(CAA)^ and tRNA^Leu(UAA)^ [188]. Additionally, the formation of m^5^C38 of tRNA^Asp^ in *S.* *pombe* and *D.* *discoideum* is stimulated by Q34 modification [189]. These modification circuits enhance the overall specificity and efficiency of modifications across the entire tRNA population, contributing to the fine-tuning of translational fidelity and cellular homoeostasis.

## tRNA modifications and regulation of tRNA-derived small RNAs

tRNA derived RNAs (tDRs), also known as tRNA fragments (tRFs) and tRNA-derived small RNAs (tsRNAs), are cleavage products from tRNA precursors and mature tRNAs. To date, more than 20,000 different tDRs have been discovered, which differ in length and sequence [190]. These tDRs have emerged as essential regulators of many biological processes, such as transposon activation, translation, innate immune responses, transgenerational inheritance, and development [191,192]. However, the rapid expansion of this field has led to confusion in their nomenclature. To address this, Holmes et al. developed the tDRnamer system, an algorithm designed to standardize the naming of tDRs [193].

tDRs are roughly classified into six different subtypes based on their length, position, and biogenesis pathways, including tRNA halves (5’-tiRNA and 3’-tiRNA), tRF-5, tRF-3, tRF-2, and tRF-1 (using a previous convention). This classification, while capturing some of their biogenesis and structural features, but remains preliminary, as their functional distinctions are still unclear. tRF-1 is derived from 3′ trailer sequences of precursor tRNAs that were digested by ELAC2 or RNase Z [194]. tRF-5, tRF-3, tRF-2, and tRNA halves are derived from mature tRNAs cleaved by angiogenin (ANG), Dicer, or other RNase at specific sites [195–197]. The generation of tDRs appears to be dynamically regulated by various stresses, including amino acid starvation, nutrient deprivation, oxidative stress, viral infection, and heat shock [198]. tRNA modifications (e.g. m^5^C, Ψ, Nm, and Q) play an important role in guiding tRNA cleavage, tDR production and function [73,74,84,199–201]. The development of high-throughput RNA sequencing technologies, such as AlkB-facilitated RNA methylation sequencing (ARM-seq), has enabled the detection of modifications in small RNAs derived from tRNAs, offering new insights into their regulation and function [53,202] (see the following sections for further discussion of tRNA-sequencing methods).

TRDMT1/DNMT2 is responsible for the m^5^C38 modification of tRNA^Asp^, tRNA^Gly^ and tRNA^Val^, whereas NSUN2 catalyzes m^5^C48, m^5^C49 and m^5^C50 in many tRNAs [133,203,204]. NSUN2 and TRDMT1/DNMT2-mediated m^5^C modifications stabilize tRNA secondary structures and protect them from ANG cleavage. Low levels of NSUN2 in mouse and human epidermal stem cells result in accumulation of tDRs (tiRNAs) that repress protein synthesis. In contrast, epidermal progenitors up-regulate NSUN2 to inhibit ANG-mediated tRNA cleavage and promote epidermal differentiation [205,206].

A prominent Ψ ‘writer’ responsible for stress-inducible Ψ on different RNAs is the multi-substrate synthase PUS7 [87,207,208]. Ψ modification at position 8 in tRNA^Ala^, tRNA^Cys^ and tRNA^Val^ enhances tDR (tRF-5) biogenesis, induces their binding to polyadenylate-binding protein C1 (PABPC1), destabilizes the translation-initiation complex and modulates global translation during development and leukaemogenesis [84,209].

The presence of Nm modification significantly affects physico-chemical properties of modified residues and increases tRNA stability [210]. Cm34 in tRNA^Met^, guided by C/D box snoRNPs (D97/D133), prevents site-specific cleavage by ANG [73]. Loss of fibrillarin, the snoRNA-guided 2′-O-methyltransferase, induces global upregulation of tRNA fragments, demonstrating the general role of Nm modification in tRNA stability [74].

In addition, Q modification in the wobble anticodon position of tRNA^His^ and tRNA^Asn^ significantly reduces ANG cleavage of these tRNAs [211]. Loss of m^5^U54 due to defective TRMT2A enhances ANG-mediated tRNA cleavage near the anticodon, resulting in the generation of tRNA halves from tRNA^Gly^ and tRNA^Glu^, further affecting cellular stress response [77]. tRNA demethylase ALKB homolog 1 (ALKBH1) and ALKBH3 can demethylate m^1^A58 modifications in tRNAs (e.g. tRNA^Met^ and tRNA^Gln^) and promote accumulation of tRNA halves by increasing tRNA cleavage by ANG [212,213].

Altogether, tRNA modifications impact structural stability and their affinity for tRNA ribonuclease. Specific tRNA modifications can govern the generation of different tDRs, impacting their role in gene regulation, stress responses and other cellular processes [214]. However, the extent to which these modifications play a role in tRNA fragment biology remains an intriguing question that is only beginning to be explored [10].

## Detection technologies for tRNA modifications

Comprehensive analysis of tRNA modifications presents unique technical challenges due to the great chemical diversity of modifications, their high density, variable stoichiometry, and the complexity of tRNA modification pathways (e.g. redundancy and genetic compensation). Ideal technologies should enable global measurements of all modifications across different classes of RNAs expressed at different levels and simultaneously achieve single nucleotide resolution and reveal their stoichiometries. While significant progress has been made in the last few years, challenges remain. Most methods developed so far have not been able to achieve these ideal goals. Here we briefly describe these methods and highlight their limitations. According to the detection principles, existing tRNA modification detection technologies can be categorized into three classes: mass spectrometry, next-generation sequencing (NGS), and nanopore-based direct RNA sequencing.

## MS-based methods

MS-based methods have been used for a very long time [215,216]. Based on the distinct chemical properties of modified nucleotides in tRNAs from their originals, liquid chromatography – tandem mass spectrometry (LC–MS/MS) has been developed as a quantitative technique for RNA modification detection and quantification [217–223]. Current protocols of RNA modification detection by LC-MS/MS are largely derived from the work pioneered by the McCloskey lab [220,221]. Briefly, tRNA molecules are digested to oligomers or monomers, and then separated by chromatography. These separated nucleosides are ionized and further fragmented into specific product ions via mass spectrometry. The mass spectrometer measures the integration of retention time, ionization properties and mass-to-charge ratio (m/z), providing information about their molecular weight and modification profile [57,224–226]. LC-MS platform provides highly precise quantification of the spectrum of modified ribonucleosides in tRNA from any organism [227].

Recent developments in highly sensitive and accurate LC-MS/MS methods allow the detection of modified nucleosides at a broad range of levels, from picogram to femtogram of modified nucleoside, achieving attomole sensitivity. To differentiate the positional isomers of modified nucleosides, which exhibit identical m/z values for molecular and nucleobase ions (e.g. m^1^A, m^6^A, m^2^A or m^3^C, m^5^C, m^4^C), Rose et al. developed an ion mobility-based MS [228]. This approach employed the differences in shape or cross-sectional area as an additional separation tool to distinguish the isomers. Another approach employed higher-energy collisional dissociation (HCD), a fragmentation technique that generates more informative MS/MS spectra of nucleosides, allowing for the identification of specific isomers of modification within a mixture [229]. In addition to providing qualitative insights, LC-MS/MS approaches can also determine the absolute amounts of modifications. For example, the triple quadrupole mass spectrometer (QQQ) is particularly well-suited for precise quantification [230,231]. Moreover, specific stable isotope labelled internal standards (SIL-IS) were used to create calibration curves for the quantification of modified ribonucleosides [232,233].

In addition to single nucleotides, MS can also be used to analyse RNA oligomers from digested tRNA [227,234,235]. Some of these digested fragments can be mapped to one or several tRNA regions, thus providing the sequence specificity required for detailed modification analysis [235,236]. For example, Limbach and colleagues developed a bottom-up RNA modification mapping approach, following RNase U2 [237], RNase MCI [238], and RNase cusativin [239] digestion of target RNAs. The Breuker group explored the top-down MS/MS approach to map RNA modifications without enzymatic hydrolysis [240]. Additionally, the Suzuki group developed the Chaplet Column Chromatography (CCC) method [241], which uses a complementary DNA to isolate target RNA for LC-MS analysis. This technique was further refined to the reciprocal circulating chromatography (RCC) [242] system. Meanwhile, a number of computational tools, such as simple oligonucleotide sequencer (SOS) [234], oligonucleotide mass assembler (OMA) and oligonucleotide peak analyser (OPA) software [243] and RoboOligo [244] have been developed to facilitate RNA modification mapping.

Moreover, LCMS-Seq was developed in 2015 to bidirectionally sequence the noncanonical RNA through a two-dimensional analysis of mass chromatograms, providing a simple and robust approach to the detection and quantification of modified RNA [245]. The two-dimensional gel electrophoresis (2D-PAGE) is a powerful technique for separating individual tRNA isoacceptors from complex mixtures of tRNAs [246,247]. In the first dimension, tRNAs are heat-denatured due to the dual action of gel temperature (70°C) and urea (8 M), and they are separated based on their length. In the second dimension, the urea concentration is reduced to 4 M, and the gel is run at room temperature. Under these conditions, tRNAs are partially refolded, and separation is based on their partial secondary conformation. Wolff et al. applied 2D-PAGE followed by mass spectrometry to analyse tRNA modifications across 79 cellular tRNAs, identifying typical modifications at previously uncharacterized positions [248]. By introducing the two-dimensional hydrophobic end-labelling strategy, Zhang et al. further developed 2D HELS MS Seq that can be used to directly analyse multiple-base modifications in RNA mixtures and enhance sample usage efficiency [249]. In 2021, Yuan et al. developed a de novo MS ladder complementation sequencing approach (MLC-Seq) that circumvents the perfect ladder requirement for direct MS sequencing, allowing de novo MS sequencing of full-length cellular tRNAs, together with all nucleotide modifications, at single nucleotide stoichiometric precision [250].

LC-MS/MS takes advantage of the physical and chemical properties of RNA, generating high quality and reproducible data. However, MS-based methods require highly specialized expertise and equipment, considerable amounts of materials, and is not suitable for high-throughput global analysis of RNA modifications. In addition, purification and enrichment protocols need to be optimized to obtain true abundance levels in biological and clinical samples [223,251].

## NGS-based methods

Sequencing-based methods are highly sensitive and suitable for detecting several tRNA modifications from small numbers of cells and clinical specimens, although these methods are applicable only to specific tRNA modifications that can be detected by sequencing, either directly, or after chemical/antibody/enzyme treatment. Current NGS-based technologies can be further classified into three types: direct sequencing, chemical-assisted sequencing, and antibody/enzyme assisted sequencing.

**Figure 3.**
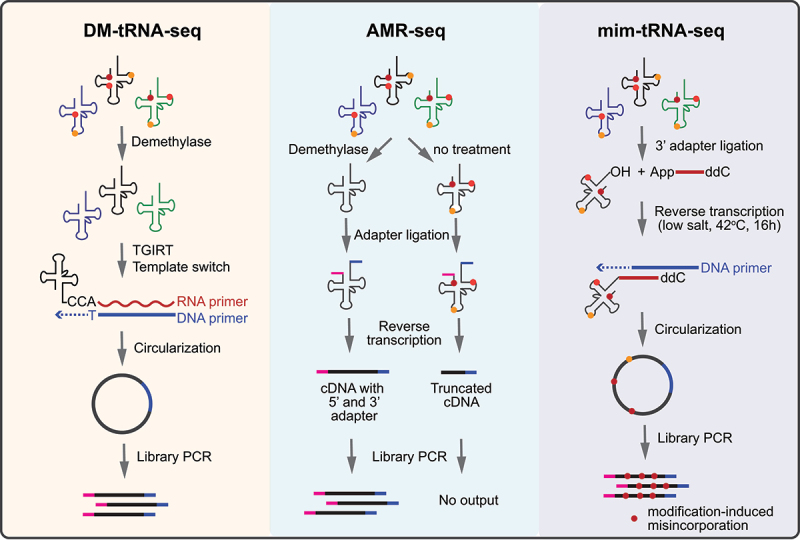
Schematic representation of direct sequencing methods for tRNA and their modifications. DM-tRNA-seq [252] uses an *E.* *coli* AlkB demethylase mixture and TGIRT reverse transcriptase in library preparation, overcoming modification and tRNA structure biases during cDNA synthesis. In ARM-seq [53], demethylase treatment enables identification of some methylated nucleosides in tRNAs, such m^1^A, m^3^C, m^1^G, and m^2^_2_G. Mim-tRNA-seq [253] uses TGIRT with optimized conditions to read through tRNA modifications and map the tRNA modifications based on modification-induced mutations.

For some modified nucleotides, the presence of chemical modifications in tRNA nucleotides can alter canonical Watson–Crick base pairing during reverse transcription (RT), leading to errors in cDNA (e.g. truncation and misincorporation). Therefore, these modification types cannot be directly sequenced without special treatments. In the 2010s, **DM-tRNA-seq**, **ARM-seq**, and **mim-tRNA-seq** were developed for the generic purpose of tRNA sequencing, but can also be used to map m^1^A, m^3^C, m^1^G, m^2^_2_G, and m^3^U modifications, in which methylation-dependent mutation signals can be detected by sequencing. At the same time, tRNA samples treated by WT/mutant AlkB demethylase are used as negative controls to reduce false-positive sites feature (Figure 3) [53,54,252–254]. The mutation signatures are not sufficient to deduce the modification identity; therefore, these methods cannot be used for de novo discovery.

**Figure 4.**
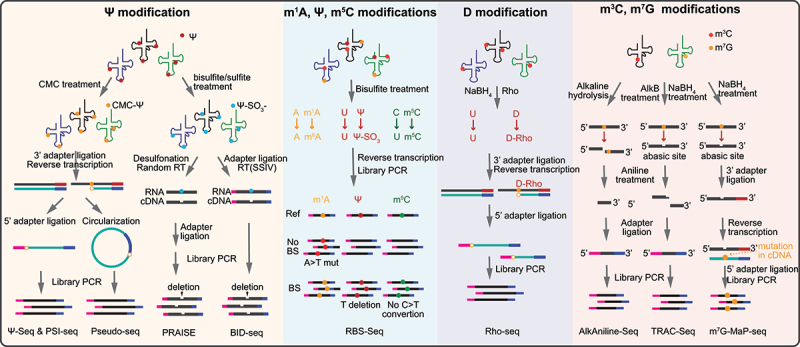
Schematic representation of chemical-assisted sequencing methods for tRNA modification analysis. Ψ-seq [207], PSI-seq [256] and Pseudo-seq [255] use CMC reaction to introduce RT stops at Ψ sites. PRAISE [257] and BID-seq [258] use a bisulphite-mediated reaction to induce nucleotide deletions at Ψ sites during reverse transcription. RBS-seq [259] utilizes bisulphite treatment to induce mutations and deletions at specific modification sites, allowing for the mapping of various RNA modifications, including m^1^A, Ψ and m^5^C. Rho-seq [260] uses NaBH_4_ and rhodamine (Rho) treatment to identify dihydrouridine (D) by capturing the RT stops caused by Rho-adducts. AlkAniline-seq [261] uses alkaline hydrolysis coupled with aniline cleavage to identify m^7^G and m^3^C sites in tRNAs. TRAC-Seq [262] utilizes borohydride reduction coupled with aniline treatment to identify m^7^G in tRNAs. m^7^G-MaP-seq [263] was also designed to identify the m^7^G modification by reading misincorporations in cDNA after NaBH_4_ treatment.

Chemical derivatization is a powerful strategy for discriminating modified nucleotides from unmodified ones based on their differential chemical properties and reactivities. For example, Ψ residues in tRNAs can be selectively labelled by N-cyclohexyl-N’-(2-morpholinoethyl)carbodiimide (CMC), and the resulting CMC-Ψ adducts stall reverse transcription, inducing truncations. Using this principle, **Ψ-Seq**, **Pseudo-seq**, and **PSI-seq** were developed to measure Ψ modification at single-nucleotide resolution [207,255,256] (Figure 4). However, CMC-based methods have low labelling efficiency and selectivity for Ψ, making it intrinsically difficult to distinguish between true Ψ signals and the background noise emerging from other bases, thus lacking stoichiometry information of Ψ.

In 2019, **RBS-seq** (a modification of RNA bisulphite sequencing) re-examined the bisulphite (BS)-mediated conversion of Ψ, and found that the Ψ-BS adduct could cause deletion signatures during RT. This involves heat-induced ribose ring opening, Mg^2+^-assisted reorientation, and base-skipping during cDNA synthesis, providing a novel way to detect Ψ (Figure 4). At the same time, RBS-Seq can detect m^5^C and m^1^A modifications that resultin signature base mismatches in bisulphite treatment conditions. RBS-Seq enables the sensitive and simultaneous detection of multiple common RNA modifications in the same RNA molecule (Figure 4) [259]. Since unmodified cytosine is also deaminated to uridine in conventional BS reaction, **BID-seq** (bisulphite-induced deletion sequencing) and similarly designed **PRAISE** (pseudouridine assessment via bisulphite/sulphite treatment) further optimized BS treatment at near neutral pH to eliminate most of the side reaction on unmodified cytosine, enabling quantitative detection of Ψ at single-base resolution [257,258].

**m^7^G-MaP-seq** (m^7^G mutational profiling sequencing) is a high-throughput approach to map m^7^G modification in tRNAs. In this method, m^7^G residues are converted to abasic sites upon sodium borohydride (NaBH_4_) treatment, leading to misincorporations during reverse transcription [264,265] (Figure 4). Similarly, the D modification in tRNA molecules can also be reductively cleaved by NaBH_4_ treatment and further labelled by a bulky rhodamine molecule (Rho), which arrests RT one nucleotide downstream of the modified position. Combining Rho labelling of the D modification with high-throughput sequencing, Rho-seq was developed to assess the presence of D at the transcriptome-wide scale and single-nucleotide resolution by comparing patterns of RT stops between NaBH_4_-treated and mock-treated samples [260] (Figure 4).

**Figure 5.**
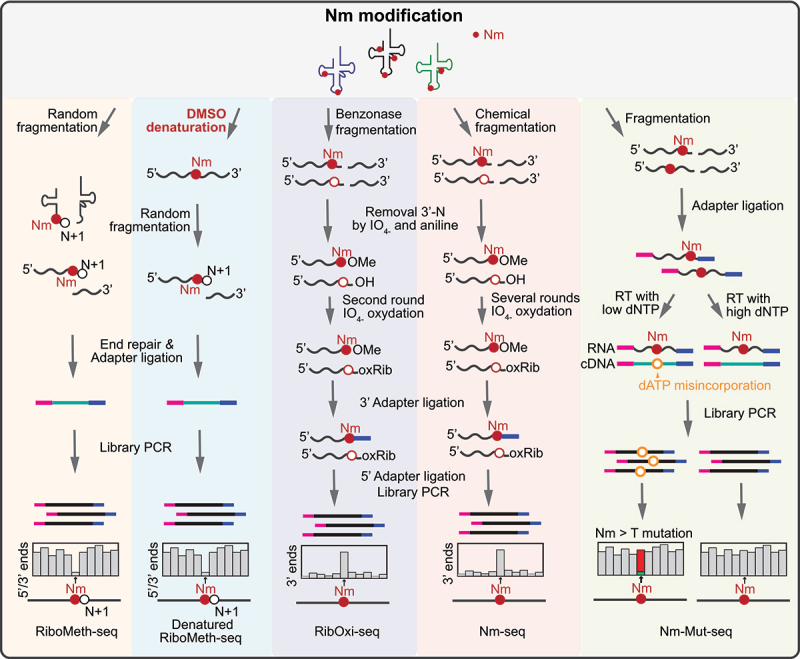
Schematic representation of sequencing methods for Nm modification in tRNAs. RiboMeth-seq [51] uses alkaline fragmentation to map Nm modification in tRNAs. Denatured RiboMeth-seq [74] (dRMS) is an improved version of RiboMeth-seq, which uses stronger denaturation conditions to improve the efficiency and uniformity of tRNA fragmentation. RibOxi-seq [267] and Nm-seq [268] use periodate oxidation-based methods for Nm detection. Nm-Mut-seq [269] is a mutation signature-based Nm mapping method that uses a newly evolved reverse transcriptase to instal mutations opposing Am-, Cm-, and Gm, but not Um.

Modified nucleotides in tRNA often exhibit distinct resistance to chemical hydrolysis compared to unmodified ones. Based on this principle, several sequencing technologies have been developed to identify RNA modifications, such as Nm, m^7^G, and m^3^C. For example, **RiboMeth-seq** was developed to map the locations of Nm within RNAs by analysing the depletion of fragment ends in sequencing, since the phosphodiester bond next to the Nm is naturally resistant to alkaline hydrolysis [51,266,267] (Figure 5). However, stable RNA structures and dense modifications strongly skew the fragmentation and reverse transcription, impeding the application of RiboMeth-seq to many ncRNAs, especially for tRNAs. To overcome this limitation, our group developed **dRMS** (denatured RiboMeth-seq), where much stronger denaturation in the presence of 95% dimethyl sulphoxide (DMSO) during fragmentation increased the efficiency, uniformity of RNA fragmentation, and detection sensitivity [74] (Figure 5).

Additionally, Nm modifications can protect the ribose against periodate (NaIO_4_) oxidation. Utilizing this mechanism, two conceptually similar methods, **Nm-Seq** and **RibOxi-Seq** (high-throughput and site-specific identification of 2’-O-methylation sites using ribose oxidation sequencing) were developed for transcriptome-wide mapping of Nm with base precision. In these methods, RNA is first randomly fragmented either chemically (using Zn^2+^ and cleavage at 95°C for Nm-Seq) or enzymatically (by benzonase for RibOxi-Seq). The RNA fragments are then subjected to 3’-terminal ribose oxidations by NaIO_4_. Nm residues at 3’-terminal resist cleavage and remain competent for the ligation of 3’-adapter, while unmodified riboses are converted to a non-ligatable dialdehyde structure. Thus, the Nm sites are detected as 3′-end peaks in sequencing reads [267,268].

Methods exploiting the steric properties of Nm also include **Nm-Mut-seq** (a mutation signature-based Nm mapping method) that uses an engineered reverse transcriptase to identify Nm sites as base mutations (including Am-, Cm- and Gm-to-T mutation) in the RT step under restrictive conditions with low deoxynucleotide triphosphate (dNTP) concentrations. However, Nm-Mut-seq is unable to detect Um in target RNA molecules [269] (Figure 5).

m^3^C and m^7^G modifications are resistant to alkaline hydrolysis, NaBH_4_ reduction and aniline-mediated cleavage. NGS-based methods like **AlkAniline-Seq** (alkaline hydrolysis and aniline cleavage sequencing) and **TRAC-seq** (tRNA reduction and cleavage sequencing) capitalize on this property by cleaving the modified sites, followed by ligation with adaptors to construct sequencing libraries for the simultaneous detection of m^3^C and m^7^G modifications [261,262,270,271] (Figure 4). Another method, **HAC-seq** (hydrazine-aniline cleavage sequencing), is designed to map m^3^C modifications in RNA molecules by hydrazine/aniline treatment, which specifically cleaves at m^3^C sites. By analyzing the distribution and frequency of cleavage events across the RNA sequence, HAC-seq provides insights into the presence and positioning of m^3^C modifications with single-nucleotide resolution [272].

To selectively enrich and map modified regions in RNA molecules, antibody-based strategies have been developed to detect several RNA modifications, such as m^7^G, m^6^A, m^6^Am, and m^5^C [263,273–275]. In these strategies, isolated RNA is first fragmented, and the modification-containing RNA fragments are enriched by specific antibody immunoprecipitation. The enriched RNAs are subjected to sequencing, and the modifications of interest can be identified by bioinformatic analysis. For instance, **m^7^G-MeRIP-seq** (m^7^G methylated RNA immunoprecipitation sequencing) is an antibody-based NGS method, which was developed to profile the global m^7^G tRNA methylome in mouse embryonic stem cells (mESCs). In addition to antibody immunoprecipitation, some enzymes or RNA modification-related proteins can also be utilized to capture modification-containing transcripts, thereby enabling the detection of some tRNA modifications [276–278]. However, a major limitation of these approaches is that they can only detect known modifications for which specific antibodies or proteins are available. Furthermore, these methods cannot provide quantitative information about modification stoichiometry and have limited sensitivity in detecting low-abundance tRNA modifications.

## Nanopore-based direct RNA sequencing (DRS)

Nanopore DRS is a unique and powerful method capable of directly analysing RNA molecules without converting them into cDNA. The first nanopore sequencing platform, MinION, was developed by Oxford Nanopore Technologies (ONT) in 2014. This technology utilizes protein nanopores to sequence single-stranded RNA by measuring ionic current changes as a set of nucleotides pass through the pore. Unlike conventional NGS methods, nanopore sequencing allows for the direct detection of RNA modifications, avoiding the biases associated with reverse transcription and PCR amplification [59,279–283]. Several other nanopore-based sequencing platforms exist, including Genia Technologies’ nanotag-based real-time sequencing by synthesis (Nano-SBS) technology, NobleGen Biosciences’ optipore system and Quantum Biosystems’s sequencing by electronic tunnelling (SBET) technology [59,284]. However, this section focuses on ONT technology, which has been the most common approach in published studies for RNA modification detection.

ONT has continually refined both the nanopore and motor proteins, which ratchets the nucleic acid molecule through the nanopore in a step-wise manner. ONT enables the direct detection of certain RNA modifications by distinguishing their current shifts from those of unmodified bases. ONT direct RNA sequencing has produced robust and reasonably high-quality data. Several pilot studies have successfully detected bulk-level RNA modifications using error distribution profiles, including tools like EpiNano [285], MINES [286], Xpore [287], Nanom6A [288] and DRUMMER [289] for m^6^A detection, ELIGOS for m^6^A and 5-methoxyuridine (5moU) [290] and nanoRMS for Ψ analysis [279]. Further advancements, including lengthening of tRNA molecules with ligated adapter extensions, have enabled tRNA molecules to be sequenced, base-called, and mapped using biological nanopores [290]. Recently, Morghan et al. developed Nano-tRNAseq, which highlighted the potential of Nanopore DRS to quantitatively detect and distinguish multiple tRNA modifications simultaneously [291], making it an invaluable tool for comprehensive tRNA modification analysis and understanding tRNA abundances and their functional roles.

However, such methods remain limited in their abilities to discover new modifications from scratch. Extensive training on known modifications and sophisticated deconvolution algorithms are required to accurately detect and interpret the presence of specific modifications. Moreover, identifying modifications in low-abundance RNA molecules is challenging. Future directions for improving nanopore DRS include enhancing the sensitivity and specificity of modification detection through advancements in nanopore technology and signal processing algorithms. Additionally, integrating nanopore DRS with other complementary techniques and developing more refined bioinformatics tools and databases will be crucial for a comprehensive understanding of the tRNA modification landscape and for discovering novel modifications.

## Disease implications of tRNA modifications

**Figure 6.**
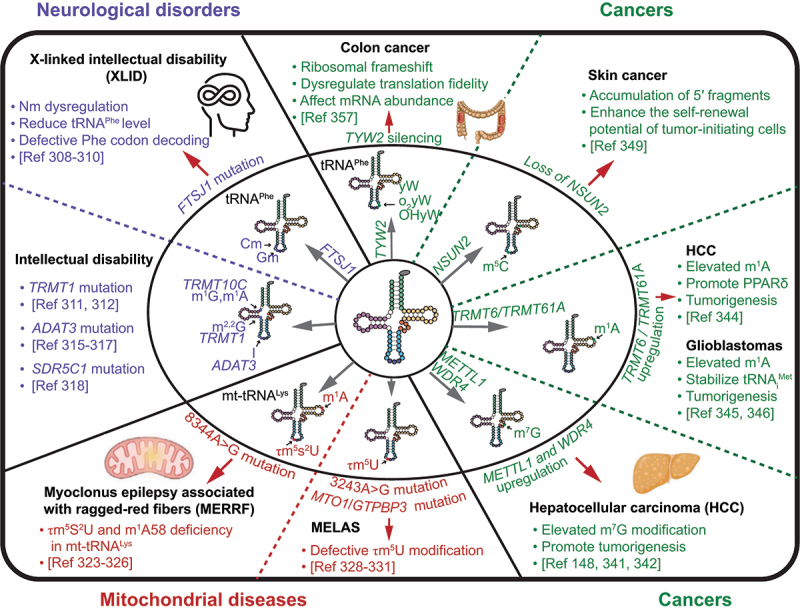
tRNA modifications and connections to human diseases. Schematic representation of the relationship between abnormal tRNA modifications and various human diseases, with a focus on neurological disorders, mitochondrial disorders, and cancers.

Dysregulation of tRNA modifications is associated with a variety of diseases due to their important functions in regulating tRNA processing, stability, and mRNA translation. Abnormal tRNA modifications are frequently found in neurological disorders, mitochondrial-linked disorders, as well as cancers (see in-depth reviews in [13,48]) (Figure 6).

## tRNA modifications in neurological disorders

Familial dysautonomia (FD) is an inherited genetic disorder of the nervous system affecting the autonomic and sensory neurons [292,293]. Numerous studies have reported FD-causing mutations in genes encoding subunits of the elongator complex [294–296]. Elongator is a six-subunit complex required for a family of multiple tRNA modifications, including mcm^5^U, mcm^5^s^2^U, and ncm^5^U. Defects in the elongator complex in FD patients result in decreased mcm^5^s^2^U at position 34 in cytoplasmic tRNAs, leading to inefficient translation, abnormal cellular processes, and neuronal dysfunction [297–299]. Disruption of the elongator complex, caused by the loss of ELP1 or ELP2, also leads to severe cardiovascular developmental defects and embryonic lethality [300,301], highlighting the essential role of mcm^5^U34 in animal development.

PUS3 targets tRNAs for Ψ modifications and its loss of function has been linked to a rare neurodevelopmental disorder [302,303]. Recently, a novel homozygous truncating mutation in PUS3 was identified in patients with intellectual impairment, who exhibited reduced levels of uracil isomerization at tRNA positions 38 and 39 [304]. Similarly, PUS7 modifies tRNAs as well as other noncoding RNAs and mRNAs. Patients with loss-of-function variants in PUS7 exhibit intellectual disability, global developmental delay, aggressive behaviour, postnatal microcephaly, and growth impairment [305,306].

FTSJ1, a homolog of yeast tRNA methyltransferase TRM7, methylates the ribose 2’-OH at positions 32 and 34 within the anticodon loop of cytoplasmic tRNAs [307]. FTSJ1 is required for expression of genes involved in neurological functions and plays a critical role in neurogenesis. Loss of function mutations in FTSJ1 are associated with X-linked intellectual disability (XLID), where they selectively reduce steady state level of tRNA^Phe^ in the brain and impede the decoding of phenylalanine (Phe) codons [308–310] (Figure 6).

Other tRNA modification enzymes that are linked to intellectual disability include the tRNA methyltransferase TRMT1 [311,312] and NSUN2 [313], thiouridylase CTU2 [314] and the adenosine deaminase ADAT3 [315]. Human TRMT1 encodes an RNA methyltransferase responsible for catalysing the m^2^_2_G modification in tRNAs. Missense mutations in TRMT1 perturbs its tRNA modification activity and causes a deficiency in m^2^_2_G formation, resulting in developmental delay, intellectual disability, and epilepsy [311,312] (Figure 6). ADAT3 encodes an enzyme that catalyze the formation of inosine (I) at the wobble position of tRNAs. Inosine enables a single tRNA to recognize and decode multiple synonymous codons, due to its ability to base pair with C, U and G. Patients with ADAT3 mutations exhibit intellectual disability, with the majority also displaying growth delay and microcephaly [315–317] (Figure 6). SDR5C1 is a member of the short-chain dehydrogenase/reductase superfamily, interacting with TRMT10C to catalyze the N^1^-methylation at position 9 of mitochondrial tRNAs, stabilizing the tRNA structure. Missense mutations in SDR5C1 leads to structural and functional alterations of tRNA, reducing mitochondrial RNA processing and modification and causing mitochondrial dysfunction and the HSD10 syndrome [318] (Figure 6). Together, a growing number of studies show the crucial roles of tRNA modifications in regulating neuronal development, providing new clues to the neurological functions of tRNA modifications.

## tRNA modifications in mitochondrial diseases

Mitochondrial diseases encompass a group of genetic disorders that are characterized by defects in mitochondrial functions caused by pathogenic mutation in mitochondrial DNA (mtDNA) or nuclear-encoded modification enzymes [319,320]. Dysregulation of mitochondria strongly impacts several organs that strongly depends on oxidative phosphorylation for energy production, such as the brain, heart, and skeletal muscles. Most diseases in this class are maternally inherited, though they can occasionally arise from de novo mutations.

Myoclonus epilepsy associated with ragged-red fibres (MERRF) [321] and mitochondrial myopathy, encephalopathy, lactic acidosis, and stroke-like episodes (MELAS) [322] syndromes are two human mitochondria-linked diseases caused by mutations in mitochondrial tRNAs (mt-tRNAs) and aberrant mt-tRNA modifications. The 8344A>G mutation in the mt-tRNA^Lys(UUU)^ gene is associated with MERRF syndrome [323]. Human myoblasts with MERRF mutations exhibit τm^5^s^2^U and m^1^A58 modification deficiency in mt-tRNA^Lys(UUU)^ [324]. Hypomodified mt-tRNA^Lys(UUU)^ lacking τm^5^s^2^U is unable to decode either AAA or AAG codon, leading to defective mitochondrial translation and respiratory activity [325,326] (Figure 6). In addition, mutant mt-tRNA isolated from individuals with MERRF exhibits reduced t^6^A37 modification of mt-tRNA^Lys^, impairing its ability to accurately decode lysine codons in mitochondrial protein synthesis [327]. Dysregulation of m^1^A58 modification in mt-tRNA^Lys^ can also affect translation elongation and the stability of nascent chains.

About 80% of individuals with MELAS have a 3243A>G mutation in the mt-tRNA^Leu(UUR)^ gene and lack τm^5^U (5-taurinomethyluridine) modification [328,329] (Figure 6). τm^5^U at position 34 (τm^5^U34) stabilizes U:G wobble base pairing on the ribosomal A-site, allowing mt-tRNA^Leu(UUR)^ to decode UUA and UUG codons. However, the hypomodified mt-tRNA^Leu(UUR)^ missing τm^5^U is able to decode UUA, but fails to efficiently decode UUG [330,331]. These data reveal the essential roles that mis-regulated mt-tRNA modifications play in mitochondrial diseases.

Moreover, several other genes are reported to be involved in mt-tRNA modifications and their dysregulation leads to mitochondrial diseases. MTO1 encodes a subunit of the enzymatic complex catalysing the modification of uridine to τm^5^U in mitochondrial tRNA. Mutations in the MTO1 gene are associated with hypertrophic cardiomyopathy and lactic acidosis. Loss-of-function mutations in MTO1 leads to dysfunction of the respiratory-chain complexes by disrupting the τm^5^U modification and impaired mitochondrial protein synthesis [332,333]. GTPBP3 encodes another subunit of the enzymatic complex responsible for the τm^5^U modification. Loss-of-function mutations in GTPBP3 gene also reduce τm^5^U modification, leading to severe mitochondrial translation defects and the development of lactic acidosis [334]. The mutations in MTU1 gene and subsequent reduction of the τm^5^s^2^U (5-taurinomethyluridine-2-thiouridine) levels in the mitochondrial tRNAs can lead to dysregulation of mitochondrial protein synthesis and mitochondrial dysfunction [335,336]. Together, these findings underscore the critical roles of mt-tRNA modifications in mitochondrial diseases and highlight the importance of understanding these modifications for developing potential therapeutic strategies.

## tRNA modifications in cancers

Cancer is one of the most complex and heterogeneous human diseases due to the large variety of genetic mutations and interactions with environmental factors. The earliest work on the roles of tRNA modifications in cancer dates back to the 1960s, when a number of tumours were found to exhibit a hypermethylation phenotype [337–339]. Srinivasan and Borek proposed this famous hypothesis in 1964: ‘Methylating enzymes as possible natural oncogenic agents’ [340], highlighting the potential role of DNA and RNA (especially tRNA) methylation in cancer formation. However, interest in cancer tRNA modifications waned as the first oncogenes were discovered in the 1970s. While DNA methylation was firmly established as a key player in cancer, tRNA methylation was largely ignored since then. However, recent years have witnessed a revival of this old hypothesis, as growing evidence suggests that many tRNA modifications and their associated writer and eraser enzymes are associated with cancer. Even though mechanistic insights remain limited to a few modifications and their associated enzymes, these studies open a new direction in cancer research. In this review, we focus on the relationship between several common types of tRNA modifications and cancer, including m^7^G, m^1^A, m^5^C, mcm^5^U34, and yW.

The m^7^G tRNA modification and the expression of its methyltransferase complex components (METTL1 and WDR4) were significantly elevated in hepatocellular carcinoma (HCC) [341,342] (Figure 6). The elevated m^7^G tRNA modification promotes the overall translation of target mRNAs with higher frequencies of m^7^G-related codons, inducing the proliferation, migration, and invasion of hepatocellular carcinoma cells [341]. Reduced tRNA m^7^G modification on tRNAs by knocking down of METTL1 severely impaired the translation of the tumour-related genes and pathways, including cell-cycle regulator Cyclin D3 (CCND3) [148], epidermal growth factor receptor (EGFR) [341], phosphatidylinositol 3-kinase (PI3K) and protein kinase B (AKT) [343]. In addition, the upregulation of the METTL1/WDR4 complex and m^7^G levels in tRNA were also shown to promote the development and malignancy of head and neck squamous cell carcinoma (HNSCC) by regulating a subset of oncogenic transcripts, including genes related to the PI3K/AKT/mTOR signalling pathway [152].

The m^1^A58 in tRNAs is catalyzed by the TRMT6/TRMT61A complex, both of which are expressed at higher levels in higher grade HCC tumours [344] (Figure 6). Elevated m^1^A promotes liver tumorigenesis by enhancing the translation of peroxisome proliferator-activated receptor δ (PPARδ), leading to higher levels of cholesterol biogenesis and activation of Hedgehog signalling [345]. More specifically, the TRMT6/TRMT61A complex instals m^1^A58 in the initiator methionine tRNA (tRNA_i_^Met^). The elevated expression of TRMT6/TRMT61A and m^1^A58 modification in glioblastomas stabilize tRNA_i_^Met^ to enhance the translation of a subset of mRNAs encoding tumorigenic proteins [346]. Moreover, the m^1^A erasers ALKBH1 and ALKBH3 promote cancer cell proliferation, migration and invasion by enhancing protein synthesis and increasing the generation of tRNA-derived small RNAs through demethylation of m^1^A in tRNA in prostate cancer [177,212].

The m^5^C modification in tRNA molecules, catalyzed by NSUN2, is crucial for maintaining tRNA stability, structure, and codon recognition [133,205,347,348]. Loss of Nsun2 enhances the self-renewal of tumour-initiating cells and contribute to the development or progression of skin tumours, suggesting a potential tumour-suppressive role for Nsun2 in skin cancer [349] (Figure 6).

AlkB homolog 8 (ALKBH8) is responsible for catalysing the formation of mcm^5^U/mcm^5^s^2^U modifications at the anticodon wobble uridines of specific tRNAs, such as tRNA^Lys(UUU)^, tRNA^Gln(UUG)^, and tRNA^Glu (UUC)^ [350–353]. ALKBH8 is highly expressed in human bladder cancers [354]. Silencing of ALKBH8 induces apoptosis and downregulation of NADPH oxidase 1 (Nox1) dependent reactive oxygen species, whereas *in* *vivo* it significantly suppresses invasion, angiogenesis, and growth of human urothelial carcinoma cells [354]. Recessive truncating mutations in ALKBH8 are also associated with intellectual disability by disrupting the mcm^5^U/mcm^5^s^2^U modifications and protein synthesis [350].

The tRNA-yW Synthesizing Protein 2 (TYW2) catalyzes Wybutosine (yW), hydroxywybutosine (OHyW) and peroxywybutosine (o_2_yW) modifications at position 37 in tRNA^Phe^, contributing to efficient codon recognition and prevention of ribosomal frameshifting events [355,356]. The unique modifications of tRNA^Phe^ are likely due to the corresponding slippage prone UUU and UUC codons. In human colon cancer, TYW2 is epigenetically silenced through DNA hypermethylation of its promoter region, inducing guanosine (G37) hypomodification in tRNA^Phe^ (Figure 6). Loss of G37 yW induces −1 ribosomal frameshift, thereby destabilizing mRNAs through nonsense-mediated RNA decay (NMD) [357]. One particular example of affected genes is the tumour suppressor Roundabout 1 (ROBO1). Loss of G37 yW in tRNA^Phe^ correlates with poor overall survival in patients with early-stage colorectal cancer through dysregulation of translation fidelity and mRNA abundance.

Overall, tRNA modifications, written by various enzymes, significantly influence tRNA’s biological functions. However, mechanistic analysis remains incomplete. In addition, some tRNA-modifying enzymes may have noncanonical functions beyond tRNA modifications, and mutations in these enzymes could affect other cellular processes. It remains unclear whether the human pathologies associated with tRNA modifications are due to altered mRNA translation or other uncharacterized mechanisms, highlighting the need for further research to elucidate the underlying mechanisms.

## Conclusions and future perspectives

tRNA is a crucial adaptor molecule that can recognize and bind mRNA codon to translate genetic information from mRNA into proteins. Unlike mRNA modifications, modifications on tRNAs are much more diverse, abundant and densely distributed. Proper tRNA modifications are fundamental for decoding the genetic code and stabilizing tRNA structures. More importantly, dynamic modifications impart regulatory roles to tRNAs, on top of their house-keeping functions. New concepts and technologies are fuelling rapid progress in this field; however, major questions and challenges remain.

First, mapping the complex tRNA modification patterns and combinations with high precision, resolution, sensitivity and stoichiometry is essential for moving the field forward. Despite the significant advancements in new technologies for mapping modifications, we are still far from achieving these goals [358]. Nucleoside analysis by LC-MS/MS is a commonly used approach and to quantify tRNA modifications in total tRNA fractions. However, MS requires considerable amounts of materials and is not suitable for high-throughput analysis of RNA modification positions. In recent years, a series of sequencing-based methods and bioinformatic procedures have been developed for RNA modification detection and quantification. Although NGS-based methods are highly sensitive for detecting tRNA modifications from small amounts of cells, they require an initial conversion of the tRNA molecules into cDNA using highly processive reverse transcriptase enzymes and/or demethylase cocktails to bypass the modifications that disrupt the Watson–Crick base pairing. Additionally, it remains challenging to integrate the detection of multiple tRNA modifications into a single sequencing-based approach. Nanopore-based sequencing offers a unique approach for directly analysing both tRNA modifications and tRNA abundances without the need for reverse transcription or PCR. With improvements in base call accuracy for RNA modification in the future, this technology has the potential to become the most promising method for detecting and quantifying tRNA modifications at a single-molecule level.

In addition to the tRNAs, it is also important to analyse modification patterns in tDRs. Recent reports have highlighted the role of tRNA modifications in the generation of tRNA-derived fragments [84,212]. Notably, tDRs have been shown to inhibit tRNA modifying enzymes, suggesting a feedback mechanism between tRNA modification and tDR biogenesis [359]. tRNA fragments are likely to carry distinct modification combinations compared to their parent tRNAs. However, few studies have analysed modifications within tDRs or their functions. Advancing NGS and nanopore-based methods, specifically tailored for small RNAs like tDRs, is essential for deeper exploration of tDR biology.

Modification mapping should be extended from simple cell lines to various tissues, developmental stages, disease states, and evolutionary contexts to understand their regulatory mechanisms. In addition to the modification chemistry, further identification and mechanistic understanding of their writers, readers and erasers are urgently needed. For example, even though we recently revealed nearly all cytoplasmic tRNAs as snoRNP targets, the precise modification sites and their functional consequences remain largely unknown, except a few [73,74]. The large number of snoRNAs (>2000 in the human genome) and their diverse expression patterns pose a major challenge in the identification of their targets [360–362].

Dysregulation of tRNA modifications can disrupt cellular processes and impair protein synthesis, resulting in various diseases. Additionally, tRNA acetylation has also been reported to play an essential role in antibiotic resistance [363], and other tRNA modifications have been reported to be linked to chemoresistance in cancer patients [364]. Understanding the roles of tRNA modifications in human disease may provide new insights for disease diagnosis, prognosis, and therapeutic interventions. Recent studies indicate that tRNA methylation enzymes, such as NSUN2 [365–367], ALKBH8 [354,368] and TRMT6/TRMT61A [345], are overexpressed in various cancers. Inhibiting these enzymes can reduce cancer cell proliferation and induce apoptosis, making them promising targets for cancer therapy [368–371].

Finally, the therapeutic applications based on tRNA modification biology are not restricted to detecting and modulating the levels of tRNA modification enzymes, but also include using tRNA modification level as diagnostic markers for various diseases [204,372–374]. The discovery of tRNA fragments and their recent implications in human diseases also provide valuable insights into human disease mechanisms [375–378]. Understanding tRNA modifications in the regulation of tRNA cleavage will further clarify their roles in diseases and provide new avenues for diagnostic and therapeutic applications.

## Data Availability

Data sharing is not applicable to this article as no new data were created or analysed in this study.
